# Delayed Stanford type A aortic dissection after transcatheter aortic valve implantation with balloon-expandable valve: a case report

**DOI:** 10.1093/jscr/rjaf875

**Published:** 2025-11-05

**Authors:** Yudai Shiwaku, Tatsuya Aonuma, Yuya Kitani, Naoko Kawabata, Erika Saito, Akiho Minoshima, Toshiharu Takeuchi, Nobuhiro Mochizuki, Masahiro Tsutsui, Hiroyuki Kamiya, Naoki Nakagawa

**Affiliations:** Division of Cardiology and Nephrology, Department of Internal Medicine, Asahikawa Medical University 2-1-1-1, Midorigaoka-higashi, Asahikawa 078-8510, Japan; Division of Cardiology and Nephrology, Department of Internal Medicine, Asahikawa Medical University 2-1-1-1, Midorigaoka-higashi, Asahikawa 078-8510, Japan; Division of Cardiology and Nephrology, Department of Internal Medicine, Asahikawa Medical University 2-1-1-1, Midorigaoka-higashi, Asahikawa 078-8510, Japan; Division of Cardiology and Nephrology, Department of Internal Medicine, Asahikawa Medical University 2-1-1-1, Midorigaoka-higashi, Asahikawa 078-8510, Japan; Division of Cardiology and Nephrology, Department of Internal Medicine, Asahikawa Medical University 2-1-1-1, Midorigaoka-higashi, Asahikawa 078-8510, Japan; Division of Cardiology and Nephrology, Department of Internal Medicine, Asahikawa Medical University 2-1-1-1, Midorigaoka-higashi, Asahikawa 078-8510, Japan; Division of Cardiology and Nephrology, Department of Internal Medicine, Asahikawa Medical University 2-1-1-1, Midorigaoka-higashi, Asahikawa 078-8510, Japan; Division of Cardiac Surgery, Department of Surgery, Asahikawa Medical University 2-1-1-1, Midorigaoka-higashi, Asahikawa 078-8510, Japan; Division of Cardiac Surgery, Department of Surgery, Asahikawa Medical University 2-1-1-1, Midorigaoka-higashi, Asahikawa 078-8510, Japan; Division of Cardiac Surgery, Department of Surgery, Asahikawa Medical University 2-1-1-1, Midorigaoka-higashi, Asahikawa 078-8510, Japan; Division of Cardiology and Nephrology, Department of Internal Medicine, Asahikawa Medical University 2-1-1-1, Midorigaoka-higashi, Asahikawa 078-8510, Japan

**Keywords:** transcatheter aortic valve implantation, delayed Stanford type A aortic dissection, balloon-expandable valve

## Abstract

Transcatheter aortic valve implantation is an established treatment for severe aortic stenosis; however, rare complications such as delayed aortic dissection (AAD) can be fatal. We report a 88-year-old woman who developed a Stanford type A AAD 14 days after transcatheter aortic valve implantation with a balloon-expandable valve. Although the patient was initially discharged without complications, she later presented with chest discomfort. A scheduled computed tomography scan incidentally revealed an AAD originating at the sinotubular junction, where the valve frame contacted calcified tissue. Consequently, a surgical repair was performed. In this case, postoperative down-titration of antihypertensive agents and dual antithrombotic therapy (aspirin and edoxaban) may have contributed to the progression of the intramural hematoma and dissection. Although delayed AAD has been predominantly reported in association with self-expanding valves, this case highlights that multiple factors can contribute to AAD with balloon-expandable valves. Therefore, patients with AAD risk factors should be closely monitored.

## Introduction

Transcatheter aortic valve implantation (TAVI) for severe aortic stenosis (AS) has been shown to be safe and effective, and the number of procedures continues to rise [1, 2]. However, TAVI-associated complications occur with a measurable incidence. In particular, mechanical injury to the aortic valve complex from the transcatheter heart valve (THV) often results in poor clinical outcomes. Among these complications, Stanford type A aortic dissection (AAD) is a rare but potentially fatal complication, occurring in 0.7%–1.9% of patients undergoing TAVI [3]. Although most cases of TAVI-associated AAD occur intraoperatively or in the immediate postoperative period [4–6], only a few reports have described delayed postoperative onset of AAD. Furthermore, most reported cases of delayed AAD involve self-expanding valves [7–9]. Here, we report a rare case of delayed AAD following TAVI with a balloon-expandable valve.

## Case presentation

**Figure 1:**
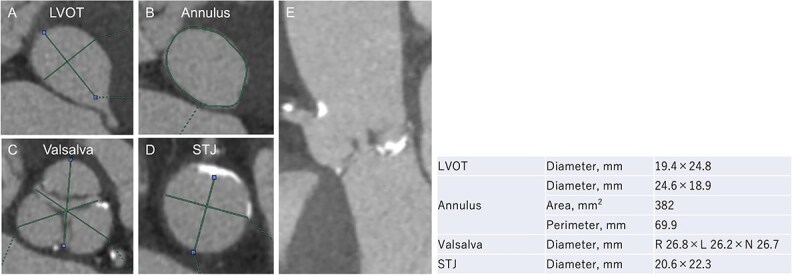
Evaluation of the aortic valve complex using contrast-enhanced CT shows axial views (A–D) and a curved multi-planar reconstruction (E) with a narrowed sinotubular junction and small calcifications (LVOT: left ventricular outflow tract; STJ: sinotubular junction).

**Figure 2:**
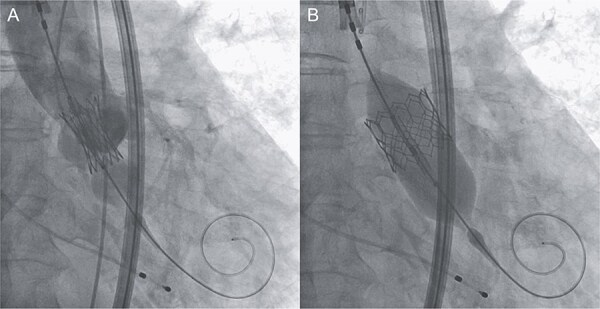
Intraprocedural angiographic images show THV implantation performed with a reduced pressure of −1 cc (A) and post-dilatation conducted with the balloon positioned toward the left ventricular side (B).

**Figure 3:**
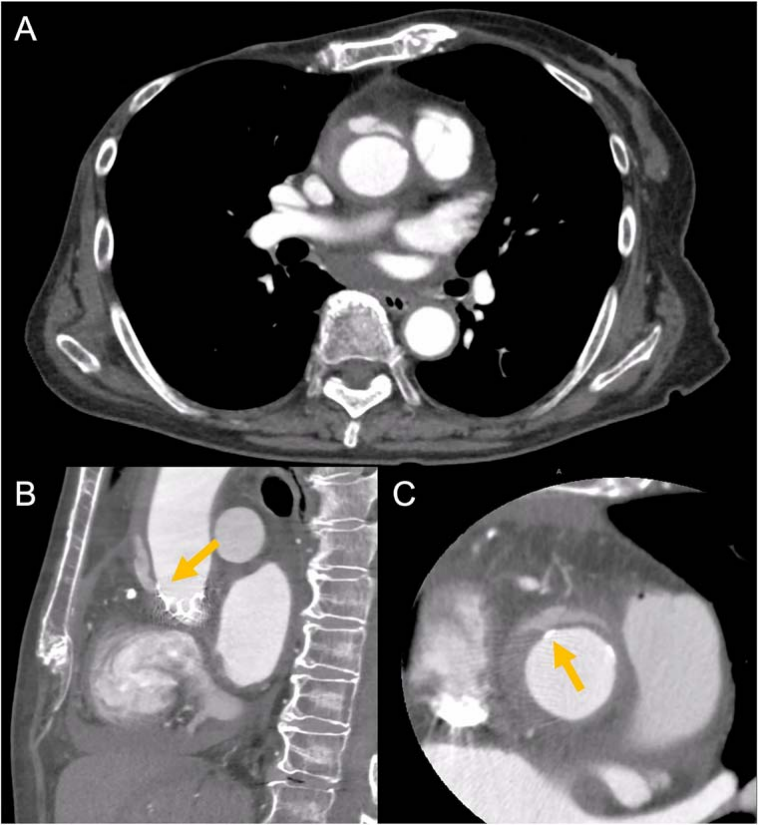
Contrast-enhanced CT on day 7 after discharge shows an AAD in the ascending aorta (A) with the entry point at the STJ extending toward the THV stent frame, suggesting an AAD associated with TAVI (B and C, arrow).

**Figure 4:**
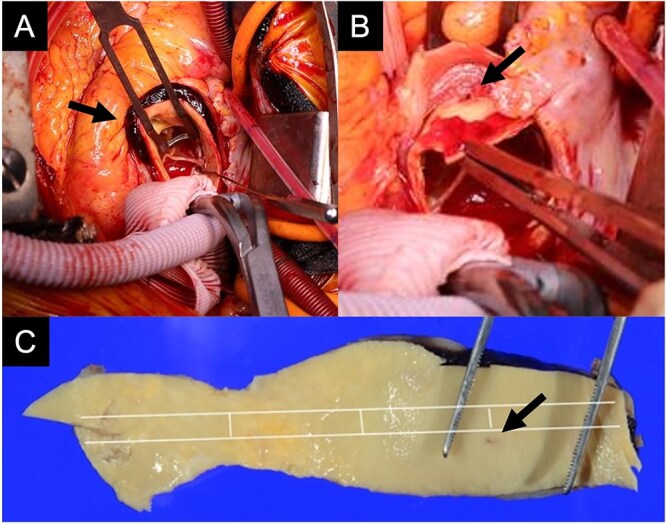
Intraoperative findings show an intramural hematoma above the ostium of the right coronary artery (A, arrow), a pinhole-like intimal defect revealed after removal of the THV and calcified tissue at the STJ (B, arrow), and macroscopic pathological examination of the resected ascending aorta confirming a small intimal defect (C, arrow).

An 88-year-old woman with severe symptomatic AS (aortic valve area: 0.40 cm^2^; peak velocity: 4.9 m/s; mean pressure gradient: 56 mm Hg) and ischemic heart disease with severe stenosis in the proximal segments of both the left anterior descending (LAD) coronary artery and circumflex arteries was referred from another hospital for invasive treatment. The patient was taking edoxaban 30 mg, nifedipine 20 mg, perindopril 4 mg, and candesartan 8 mg for the treatment of atrial fibrillation and hypertension. The patient’s perioperative risk was high, with a Society of Thoracic Surgeon (STS) score of 9.31% and Logistic EuroSCORE of 23.11%. Following discussion with the heart team, we decided to perform TAVI before coronary revascularization, with percutaneous coronary intervention (PCI) planned postoperatively. We evaluated the aortic valve complex using contrast-enhanced computed tomography (CT), which revealed a narrow sinotubular junction (STJ) with small calcifications measuring 20.6 × 22.3 mm (Fig. 1). TAVI was performed via a transfemoral approach under local anesthesia and sedation. A 23-mm SAPIEN 3 THV (Edwards Lifesciences, Irvine, CA, USA), which provides better coronary access due to its shorter frame length and relatively larger cell size, was deployed without prior balloon aortic valvuloplasty. To minimize the risk of injury to the STJ, the THV was deployed with a reduced balloon volume (−1 cc), and post-dilatation was conducted with the balloon positioned toward the left ventricular side (Fig. 2). Postoperatively, the patient’s blood pressure stabilized with rest and a reduced-sodium diet, allowing modification of the antihypertensive therapy to sacubitril/valsartan 100 mg alone. Additionally, aspirin 100 mg was added to ongoing edoxaban therapy for the planned PCI. The postoperative course was uneventful, and the patient was subsequently discharged. However, on day 4 after discharge, the patient experienced acute chest discomfort. Blood tests, electrocardiography, and transthoracic echocardiography revealed no significant changes from the time of discharge, and the patient was sent home as the symptoms gradually improved. On day 7 after discharge, contrast-enhanced CT—originally scheduled as a preoperative assessment for PCI at the referring hospital— incidentally revealed a localized dissection of the ascending aorta. The entry point of the dissection was located at the STJ extending toward the THV stent frame, consistent with Stanford AAD associated with TAVI (Fig. 3). The patient was transferred to our hospital and underwent surgery, including aortic valve replacement, ascending aorta replacement, and coronary artery bypass grafting (left internal thoracic artery to the LAD and saphenous vein graft to the obtuse marginal branch). Intraoperatively, removal of the THV and calcified STJ tissue revealed a pinhole-like intimal defect, which was continuous with a hematoma within the media and identified as the entry point of the dissection. Macroscopic pathological examination of the resected ascending aorta also confirmed the presence of a small intimal defect (Fig. 4). This intimal injury was therefore considered to have resulted from compression of the calcified tissue caused by THV implantation. After ~1 month of rehabilitation, the patient was discharged in an ambulatory state on postoperative day 30.

## Discussion

This report presents the case of an 88-year-old woman with delayed AAD following TAVI, likely due to compression of the calcified tissue by the implanted balloon-expandable valve. Although several cases of delayed AAD after TAVI have been reported, the majority have involved the use of self-expanding valves [7–9]. This case is therefore significant for its association with a balloon-expandable device.

The etiologies of AAD associated with TAVI can be divided into the following two categories: (i) procedural factors, such as device-induced injury to the aortic valvular complex; and (ii) patient-related factors, including anatomical abnormalities or conditions and medications that affect the aortic complex, such as infection, vasculitis, hypertension, or antithrombotic therapy [4–10]. The higher incidence of delayed AAD associated with self-expanding valves is thought to be related to their structural design, which is characterized by a longer longitudinal length and prolonged contact between the THV stent edge and ascending aorta. Given their gradual expansion mechanism, these valves may cause delayed mechanical injury to the aortic wall over time [9]. In contrast, most reported cases of AAD associated with balloon-expandable valves have occurred intraoperatively or in the immediate postoperative period, as the mechanical stress on the aortic wall peaks during balloon inflation [5, 6, 9]. In the present case, the intimal injury was extremely small and pinhole-like, suggesting that the dissection likely did not occur during valve implantation. However, the patient experienced acute chest discomfort after discharge, and it was presumed that AAD had occurred at that time. Postoperative down-titration of antihypertensive agents and dual antithrombotic treatment (aspirin and edoxaban) may have contributed to the progression of intramural hematoma and the delayed onset of dissection. These findings suggest that the mechanisms underlying delayed development of AAD after TAVI are multifactorial. Even with the use of a balloon-expandable valve, the risk of delayed AAD remains. In patients with these risk factors, it is important to perform intraoperative transesophageal echocardiography under general anesthesia and postoperative contrast-enhanced CT for the early detection of AAD.

## Conclusion

Delayed AAD following TAVI is a rare complication that results from a combination of procedural and patient-related factors. As demonstrated in this case, it can also occur with the use of balloon-expandable valves. Therefore, patients with relevant risk factors should be monitored closely.

## References

[1] Reardon MJ, Van Mieghem NM, Popma JJ, et al. Surgical or transcatheter aortic-valve replacement in intermediate-risk patients. N Engl J Med 2017;376:1321–31. 10.1056/NEJMoa1700456 28304219

[2] Mack MJ, Leon MB, Thourani VH, et al. Transcatheter aortic-valve replacement with a balloon-expandable valve in low-risk patients. N Engl J Med 2019;380:1695–705. 10.1056/NEJMoa1814052 30883058

[3] Thomas M, Schymik G, Walther T, et al. Thirty-day results of the SAPIEN Aortic Bioprosthesis European Outcome (SOURCE) Registry: a European registry of transcatheter aortic valve implantation using the Edwards SAPIEN valve. Circulation 2010;122:62–9. 10.1161/CIRCULATIONAHA.109.907402 20566953

[4] Brinkmann C, Koeppel T, Schofer J. Endovascular entry closure of a late type A aortic dissection after implantation of a self-expanding transcatheter heart valve (Evolut R): a case report. Eur Heart J Case Rep 2021;5:1–6. 10.1093/ehjcr/ytab343 PMC8453414 34557637

[5] Yashima F, Hayashida K, Fukuda K. Delivery balloon-induced ascending aortic dissection: an unusual complication during transcatheter aortic valve implantation. Catheter Cardiovasc Interv 2016;87:1338–41. 10.1002/ccd.25997 25963735

[6] Rujirachun P, Junyavoraluk A, Jakrapanichakul D, et al. Immediate aortic dissection after transcatheter aortic valve replacement: a case report and review of the literature. Clin Case Rep 2021;9:e04412. 10.1002/ccr3.4412 PMC8259930 34257980

[7] Pontious ME, Ashfaq A, Watson JJ, et al. Late type a dissection after transfemoral aortic valve replacement. JACC Case Rep 2020;2:877–81. 10.1016/j.jaccas.2019.12.050 34317372 PMC8302056

[8] Losmanová T, Tosoni I, Fahrni S, et al. Autopsy case of aortic dissection after transcatheter aortic valve implantation (TAVI). BMJ Case Rep 2018;2018:bcr2017220105. 10.1136/bcr-2017-220105 PMC5878380 29545421

[9] Al-Attar N, Himbert D, Barbier F, et al. Delayed aortic dissection after transcatheter aortic valve implantation. J Heart Valve Dis 2013;22:701–3. 24383384

[10] Naar J, Vondrakova D, Kruger A, et al. Cardiac arrest as an uncommon manifestation of late type a aortic dissection associated with transcatheter aortic valve replacement. J Clin Med 2023;12:5318. 10.3390/jcm12165318 37629360 PMC10455525

